# A Call to Integrate Measures of Environmental Context into Research on Maternal Brain Health

**DOI:** 10.3390/ijerph22060818

**Published:** 2025-05-22

**Authors:** Sofia I. Cárdenas, Bridget L. Callaghan

**Affiliations:** 1 Department of Psychology, University of Southern California, Los Angeles, CA 90007, USA; 2 Department of Psychology, University of California, Los Angeles, CA 90095, USA; bcallaghan@ucla.edu

**Keywords:** pregnancy, neuroscience, maternal brain, mental health, environmental context

## Abstract

Environmental factors—especially those related to interpersonal relationships and physical resources—profoundly impact women’s neurobiology and mental health. Despite this, environmental factors, including socioeconomic status, early life adversity, and neighborhood resources, are less explored within the maternal brain literature. This literature highlights pregnancy as a developmental phase in adult women’s lifespans marked by neurobiological shifts supporting fetal development and optimizing caregiving behaviors. While neurobiological shifts during this period are well-documented, pregnancy is also associated with a heightened risk for mental health challenges. This narrative review, focusing on the last 10 years, examines the research that underscores the importance of integrating environmental factors into research frameworks to comprehensively understand their effects on maternal neurobiology and mental health throughout pregnancy. Building on this research, authors discuss future research methodologies that will support a more comprehensive understanding of the intersection between environmental contexts, maternal neurobiology, and mental health.

## 1. Introduction

Pregnancy is a time in a woman’s life characterized by changes, including shifts in brain structure and biological function [1]. Critically, these changes are thought to underlie optimal parenting behaviors, such as emotion regulation and planning, maternal metabolic health, and adjustment [2]. However, it is also clear that pregnancy can be a fraught period in which mental health risks, especially, become increasingly prevalent. Specific environmental contexts may exacerbate maternal mental health issues, and such contexts may achieve these risk-increasing properties via their sculpting impact on women’s neurobiology [2,3,4]. However, research examining the impact of environmental contexts on women’s neurobiology and mental health in pregnancy is scarce. This narrative review promotes the idea that such contextual factors are critically important to incorporate into research designs to analyze maternal neurobiology during pregnancy. Insights gained from this review have clear translational implications, allowing researchers, clinicians, and policymakers to develop targeted interventions for family well-being.

The current narrative review addresses one larger, overarching objective—to examine advancements in understanding how context shapes maternal neurobiology and mental health during pregnancy. To achieve this objective, the narrative review has three components. First, we begin by taking a broad view, identifying how environmental context impacts women’s neurobiology and mental health in general. Second, we review how women’s neurobiology and mental health are affected by pregnancy. Third, we examine how ecological context impacts maternal neurobiology and mental health during pregnancy. In conclusion, we discuss future directions the field can take in measuring the influence of environmental contexts on neurobiological and mental health changes during pregnancy.

## 2. Methods

This narrative review surveys the literature on environmental context, maternal brain, and mental health, emphasizing the gestational period and the first year postpartum. The literature search was completed with PubMed and Google Scholar search queries between January 2025 and March 2025 without systematic search terms. An incomplete example of search terms includes “maternal brain”, “social determinants of health”, “early adversity”, “mental health”, “perinatal mental health”, and “poverty”. Additional literature was identified in the literature reference lists. After the literature search, papers were assessed to determine whether the articles emphasized a link between environmental context and maternal neurobiology or between environmental context and maternal mental health.

## 3. Environmental Context Impacts Women’s Neurobiology and Mental Health

Environmental context, encompassing both relationships and the physical environment, plays a vital role in women’s health. Concerning relationships (e.g., familial, romantic, caregiving), women who experience early life adversity are at heightened risk for poor health outcomes across the life span, including accelerated epigenetic aging [5], worse executive functioning in middle age ([6]), and worse physical health, including increased risk of sexually transmitted infections and increased risk of obesity [7]. Furthermore, beyond early life experiences, the act of caregiving for others has been associated with adverse health outcomes in women [8]. In particular, caring for aging spouses is linked to the onset of hypertension [8] as well as an increase in the severity of menopause symptoms [9].

Recent research has examined how physical environments influence women’s health. For example, in a recent scoping review, Perez and colleagues [4] examined social determinants—such as early life stress, socioeconomic status, and built/physical environment—of inflammatory markers linking depression and type 2 diabetes among women. Of the 46 studies, only 6 examined factors involving the physical environment and inflammatory markers in women [4]. These studies found that adult inflammation was associated with various physical environment factors, including higher levels of air pollution [10], neighborhood socioeconomic deprivation [11], and exposure to poorly maintained buildings and neighborhoods during childhood [12]. The review emphasized the complexity of measuring the physical environment, given that this factor is measured by various indicators, such as air pollution, neighborhood deprivation, neighborhood socioeconomic status, perceived neighborhood safety, and early life built environment [4]. In addition, the physical environment overlaps with other factors, such as socioeconomic status and race [4], which themselves are associated with women’s health outcomes. In another study assessing the physical environment and health, Matsumoto and colleagues ([13]) found that proximity to public transportation was associated with lower odds of depression over a three-year period in a sample of older adults (*n* = 4947). Considering the extensive evidence showing that environmental contexts—measured through relationships and the physical environment—impact women’s health, a logical next step is to consider how they impact women during a critical life stage: pregnancy.

## 4. Pregnancy Impacts Women’s Neurobiology and Mental Health

Since the late twentieth century, researchers have used neuroimaging methodologies (e.g., MRI, EEG) to understand how women’s neurobiology shifts during the transition to motherhood, with many of those changes being linked to maternal caregiving behaviors [14]. Lorberbaum and colleagues [15] conducted the first study examining brain responses in first-time mothers (*n* = 4) with infants ranging from a few weeks to 3.5 years of age. In response to infant cries compared to white noise, mothers showed greater activation in reward regions, including the anterior cingulate cortex [15]. From these early studies, more than a dozen subsequent studies with larger sample sizes and more methodological controls (e.g., age of infants) examined how new mothers respond neurally to various infant stimuli (e.g., cry sounds, pictures, videos) of their infant versus another infant [14]. These studies found that mothers show increased activation to their babies compared to unknown babies, especially in regions supporting self-awareness (i.e., insula), emotion regulation (i.e., dorsolateral prefrontal cortex), and visual processing (i.e., occipital cortex), paving the way for current theories of a “maternal neural network” [16].

Beyond functional changes, structural neuroimaging studies have found notable changes in maternal brain volume [17,18,19]. Oatridge and colleagues [19] conducted the first structural neuroimaging study examining gray matter volume from the prenatal period to postpartum in first-time mothers (*n* = 9), finding that mothers show reductions in brain volume from prenatal to postpartum and a rebound in brain volume at six months postpartum [19]. More recently, research has moved towards examining mothers’ brain structure and function changes, utilizing larger sample sizes, age-matched control groups, multiple time points across the perinatal period, and hormonal assays [18]. A longitudinal study by Hoekzema and colleagues [17] found that compared to a nulliparous control group of never-pregnant women, first-time mothers show reductions in gray matter volume from prenatal to postpartum in the cortical midline, bilateral lateral prefrontal, and temporal cortex [17]. Notably, the degree of changes predicted the mother’s self-report postpartum attachment [17]. Martínez-Garcia and colleagues [20] organized key findings from longitudinal studies on gray matter volume changes during the perinatal period, noting consistent changes in frontal and subcortical areas. Frontal areas include superior temporal and parietal gyri, precuneus, postcentral and precentral gyri, anterior cingulate cortex, and insula [20]. Subcortical areas include the hippocampus, amygdala, thalamus, caudate, nucleus accumbens, and cerebellum [20]. Few studies have examined gray and white matter changes across the perinatal period. However, Pritschet and colleagues [21] conducted a dense sampling neuroimaging study of one woman from preconception to two years postpartum, finding that in tandem with decreased gray matter volume across the postpartum period, the woman showed increased white matter microstructural integrity [21]. Hormonal fluctuations and parenting experience likely drive changes in women’s brain structure during pregnancy [18,20,22].

Research on maternal neurobiology during pregnancy provides insights into how women’s neurobiology relates to parenting behavior. For instance, in a recent longitudinal study, researchers assessed trajectories of women’s (*n* = 127) brain structure using MRI across pre-pregnancy (i.e., preconception), during pregnancy (i.e., 18 weeks gestation, 34 weeks gestation), and postpartum (i.e., 1 month postpartum, 6 months postpartum) in a sample of first-time mothers [22]. They found that gray matter volume decreased from pre-pregnancy into pregnancy in the inferior parietal, superior frontal, supramarginal, precuneus, and superior temporal regions. Furthermore, gray matter volume returned to baseline volume at postpartum. Finally, the degree of return of gray matter to baseline volume at postpartum was linked with greater attachment and reduced hostility towards the infant [22]. In a different study, using a single case study design, Pritschet and colleagues [21] recruited one pregnant woman to complete 26 MRI scanning sessions ranging from 3 weeks before conception through 2 years postpartum [21]. Somewhat similar to Servin-Barthet and colleagues [22], Pritschet and colleagues’ [21] results revealed widespread decreases in gray matter volume and cortical thickness across the brain from prenatal to postpartum, with a partial rebound at postpartum as well as increased white matter microstructural connectivity during the prenatal period and a return to baseline at postpartum [21]. In addition, concerning EEG, Patrick and colleagues [23] conducted a study assessing links between mothers’ neural response to emotional cues and parenting behavior in a sample of mothers (*n* = 137) and their six-month-old infants. Their results revealed that mothers with dampened neural patterns of facial processing (i.e., N170) to distressed infant (vs. neutral infant) faces were more likely to show intrusive maternal behavior. In addition, mothers with more significant neural signals to attention processing (i.e., P300) to happy infant (vs. neutral infant) faces were more likely to show sensitive maternal behavior [23]. Though parenting behavior is the only consistently evaluated behavioral outcome of mothers’ neurobiological adaptation, others have proposed that similar brain changes could be linked to fundamental sensory processing [2]. Thus, pregnancy research paradigms are shifting to examine pregnancy as a crucial transition in women’s long-term neurobiological and mental health with implications for many domains of women’s health and functioning across the lifespan.

Beyond neurobiological measures, research has also increasingly explored how cognition changes throughout pregnancy and postpartum. One cognitive change studied extensively during pregnancy and postpartum is colloquially known by the term “baby brain”. The term refers to memory lapses and forgetfulness self-reported by many pregnant and postpartum women [24]. In a recent systematic review of 20 studies including 709 pregnant women and 521 controls, Younis and colleagues [25] found subtle evidence that pregnancy alters women’s verbal memory and attention compared to non-pregnant control groups and that these changes may persist postnatally [25]. Across time, baby brain literature has received more methodological and theoretical scrutiny, with critics emphasizing that studies examining perinatal cognition focus on specific laboratory tasks (e.g., verbal paired associates, prospective memory), that many studies yield no differences in cognitive performance between pregnant and control groups of non-pregnant women, and that the interpretation of results highlights deficit-based models of maternal performance while ignoring adaptations [26,27]. Looking ahead to advancements in pregnancy research, Callaghan and Pawluski [26] encourage more research on cognition that integrates an adaptation-based perspective and assesses ecologically relevant material. For example, Callaghan and colleagues [28] used ecologically relevant material to the experience of motherhood to examine spatial associative memory in pregnant women during the third trimester of pregnancy (*n* = 74). They found that pregnant women, compared to never-pregnant women, showed enhanced spatial memory (i.e., retention of object–scene–location associations) and improved learning of the ecologically mothering-related stimuli (e.g., highchair, infant, bib) [28]. While maternal caregiving behavior is the most commonly assessed aspect of women’s cognitive functioning during pregnancy and postpartum, Callaghan and Pawluski [26] reimagine a broader future for this research. They emphasize scientific rigor and ask researchers to challenge stereotypes surrounding women’s cognitive abilities becoming impaired during pregnancy [26]. Though beyond the scope of this review, research on women’s cognition and hormone fluctuations during pregnancy coincides with research on women’s cognition during other periods of hormonal fluctuation, such as menopause (See [29,30]).

In addition to research on women’s cognition, a large and growing body of pregnancy and postpartum research focuses on women’s stress reactivity and mental health [1]. For instance, one study found that greater self-reported maternal perceived stress in the prenatal period was associated with greater neural attention responses to low-distress infant cries [31]. In contrast, another study showed that less reactive cortisol responses to separation from an infant were associated with greater functional brain responses in areas supporting emotion processing (i.e., limbic/paralimbic) and emotion regulation (i.e., prefrontal circuits) to infant cry versus white noise sounds during an MRI task [32]. Both aforementioned studies informed the understanding of mothers’ hormonal and neural stress reactivity patterns to parent-specific cues. Still, another reported that greater socioeconomic disadvantage (income-to-needs ratio) was linked with elevated neural activation in the amygdala to negative infant facial expressions and dampened neural activation in the amygdala to positive infant facial expressions [33]. Concerning depression, compared with healthy postpartum mothers, mothers with postpartum depression had lower cortical thickness in regions involved in empathy and sensory processing, such as the insula and parietal lobe [34], as well as altered fMRI resting state connectivity patterns in regions involved in emotion and self-referential processing, such as the hippocampus, precuneus, and superior frontal gyrus [35]. Recent clinical neuropsychological research has shown that mothers with risk factors for psychosis (i.e., bipolar disorder, schizoaffective disorder, or previous episodes of postpartum psychosis), combined with poor prenatal neuropsychological functioning, are a significant risk factor for postpartum psychosis. This finding highlights neurocognitive risk factors associated with postpartum psychosis [36]. Taken together, these studies emphasize the interplay between mental health symptoms and neurobiology during the perinatal period, emphasizing the need for a comprehensive understanding of how these elements influence maternal well-being and caregiving.

## 5. Environmental Context Impacts Women’s Neurobiology and Mental Health During Pregnancy

While much of the extant research on maternal neurobiology, cognitive and emotional health to date has focused on charting normative changes across pregnancy and into the postpartum period or using neurobiology and cognitive measures to predict mental health risk, there is a growing body of research during pregnancy and postpartum which has attempted to highlight the influence of environmental contexts—such as the physical environment and interpersonal relationships—on women’s neurobiology and mental health during this stage of adult development [33,37,38,39]. Concerning the physical environment, studies using both animal and human models emphasize the role of environmental resources in supporting mothers’ neurobiological adaptations and mental health during pregnancy [33,40]. In an experimental animal study conducted by Chen and colleagues [40], the effects of environmental enrichment on depressive-like behaviors were examined in a sample of mother rats (*n* = 28). The enriched environment included various facilities that stimulate locomotor, olfactory, and visual development, such as tree holes, floral scented balls, and colorful beads [40]. The subjects were assigned to one of four conditions: a standard environment with and without maternal separation from the litter and an enriched environment with and without maternal separation from the litter. Among the sample of mother rats with a history of maternal separation and related postpartum depression symptoms, environmental enrichment during pregnancy and postpartum significantly reduced depressive phenotypes, evidenced by reduced neuroinflammation and improved synaptic plasticity [40]. This foundational research provides insight into the mechanisms by which environmental contexts influence maternal mental health outcomes. In human studies, Kim and colleagues have identified a correlation between more significant socioeconomic disadvantage in mothers and lower functional brain response to infant cry sounds in regions that support emotion regulation (e.g., medial prefrontal cortex), emotion value (e.g., prefrontal gyrus), and sensory processing (e.g., superior temporal gyrus). Findings indicated that perceived stress, rather than depression symptoms, primarily drove the relationship between mothers’ environmental stress, caused by socioeconomic deprivation and brain changes [33]. These studies highlight the importance of examining contextual features that affect mothers in interpreting neurobiological and behavioral changes that occur during the pregnancy and postpartum stages of development. Beyond impacting mothers’ mental health and neurobiology, mothers’ socioeconomic and life circumstances likely shape fetal development. The Barker hypothesis suggests signals (e.g., nutrition, stress, substance exposure) shape fetal development and result in distinct infant phenotypes (e.g., birthweight, heart rate [41,42]). Many of these phenotypes are, in turn, linked to lasting adult health conditions (e.g., psychopathology, cardiovascular disease [42]). Building on the Barker hypothesis, further scientific understanding of environmental context on mothers’ mental health and neurobiology has implications for the long-term health of children.

Another important contextual feature to consider in interpreting neurobiological changes during pregnancy and postpartum concerns individual differences in the social environments in which mothers live. Indeed, social relationships and connectedness are linked to reduced all-cause mortality and various health morbidities [43]. A notable meta-analysis involving 64,449 women from 67 articles found that low social support significantly predicted an increased risk of mental health symptoms (e.g., depression, stress, and self-harm) during pregnancy [44]. Beyond close relationships, being in a community aligned with one’s cultural identity can support mental health during pregnancy. For example, Luis Sanchez and colleagues [38] examined acculturation stress on postpartum depression symptoms in a sample (*n* = 159) of pregnant American women of Mexican descent. Their results revealed that more of an increase in orientation to one’s Mexican cultural identity from prenatal to postpartum predicted fewer postpartum depression symptoms [38]. For more insights on this topic, Dunkel Schetter and colleagues [45] and Ramos and colleagues [46] provide in-depth discussions of the role of cultural context on mothers’ mental health and neurobiology during the perinatal period [45,46].

Beyond current social relationships and cultural identity, early life experiences from childhood may play a role in women’s adaptation to pregnancy and parenting. Specifically, interoception, which refers to the neural process of perceiving and modeling sensory information from the body, has been proposed to improve during pregnancy [2,47]. In that theoretical paper, Savoca and colleagues suggested that the neural changes reported to occur during pregnancy, along with the influence of surging neurosteroids associated with pregnancy, would likely result in improved interoception via acting on the excitatory–inhibitory balance in the brain. However, they suggested that this improvement in interoception during pregnancy might be moderated by mothers’ exposure to childhood adversity. In line with their hypotheses, self-reported interoception has been shown to strengthen across gestation and into the postpartum period [48], be higher in pregnant women than non-pregnant women, and be linked with antenatal attachment [49]. Also, interoception has been shown to increase with parity [50]. However, building on existing research linking both lower interoception and higher early life adversity with increased depression risk, Savoca and colleagues [2] examined the role of interoception ability and early life adversity on depression risk during pregnancy [2]. As expected, Savoca and colleagues [47] found that early life adversity was associated with lower self-reported interoception ability in their sample of pregnant women. In addition, the degree to which higher interoceptive ability was linked with reduced depressive symptoms was associated with the amount of early life adversity exposure.

A final environmental factor to consider relates not to the environments in which women themselves are living and affected, but those that directly influence the scientists conducting this important research on pregnancy and postpartum brains and behavior. Despite advancements in women’s research on pregnancy, some theorize that current knowledge is limited partly due to the infrastructure of women’s health research [51]. For instance, African American and Black scientists receive fewer National Institutes of Health R01 grants, driven by lower discussion rates, lower impact score assignments, and topic choices classified as lower scientific priority [52]. Who is involved in maternal health research will influence the research choices and, thus, what we learn about this critical stage of life. As one potential reflection of these diversity issues, a recent meta-analysis of 18 studies showed that only one study examined race as a potential moderator of mothers’ neural responses to infant stimuli [39,53,54]. In some respects, these sampling disparities may be driven by the funding inequities mentioned earlier. An additional concern is the methodological limitations of neuroimaging methodologies, which may bias who can be involved in women’s health research from the participant’s end. Clinical standard electrodes used in EEG/ERP research have lower signal-to-noise ratios in women’s hairstyles from non-White/European ancestry, resulting in systematic sampling bias in study samples [55]. As such, it is not only the range of environmental factors impacting women who are participating in research that is important to study, but it is also worth considering that environments influence who is doing the research and how it is done, shaping what is known and can be known about the maternal brain. While the research on the maternal brain continues to reveal important insights into this fascinating stage of adult development, a concerted effort needs to be made to understand the numerous environmental factors that contribute to individual differences in the adaptation to pregnancy and parenting [56].

## 6. Future Directions in Measuring Environmental Context During Pregnancy

One proposed pathway for integrating measurement of environmental context into maternal brain research would be to make increased use of multi-site studies, building on epidemiological research to investigate a range of environmental factors that influence women’s brain health and mental well-being during pregnancy [3,56]. These studies can incorporate relevant environmental contexts, such as neighborhood risk and air pollution, and identify population-level factors, such as health-seeking behavior and access to health care, impacting women’s brain and mental health during pregnancy. In addition to understanding population-level health-seeking behaviors and access to care, epidemiological studies may provide insight into the consequences of historical inequity on health behaviors during the perinatal period.

Building on epidemiological studies, multi-site studies may provide opportunities to examine environmental factors shaping women’s brains and mental health during pregnancy while integrating relevant environmental context aspects (e.g., neighborhood risk, air pollution). In 2016, the National Institutes of Health began the Environmental Influences of Child Health Outcomes (ECHO) study, a consortium of 30 cohorts across multiple sites [57]. The ECHO-PATHWAYS Consortium is a multi-site national program fostering collaboration across various studies to address core child-health challenges, including neurodevelopmental disorders. Using multi-sites, the ECHO-PATHWAYS Consortium has amassed the largest birth cohort in the United States, including 50,000 mother–child dyads. Researchers in the ECHO-PATHWAYS Consortium have worked to “harmonize” or standardize measures of environmental context that shape child outcomes to maximize both sample size and generalizability of findings across all studies. Looking ahead, research on women’s brains and mental health during pregnancy should consider a multi-site framework to allow for pooling data across pregnancy cohorts and maximizing the generalizability of findings. Several studies to emerge from the ECHO-PATHWAYS Consortium already support the view that integrating measures of environmental context helps promote understanding of women’s outcomes during pregnancy [58,59,60]. In the future, consortium models focused on environmental context and maternal health can support the collection of large-scale, diverse samples from various backgrounds, thereby enabling more externally valid models of maternal health.

In addition to large-scale consortium studies, advancing understanding of the environmental factors influencing maternal health requires dense sampling studies with smaller, more focused samples. Dense sampling studies, such as those led by Dr. Emily Jacobs, have provided novel insight into how a woman’s brain tissue structure and hormones fluctuate over time [21,61]. By following individuals over extended periods, these studies can capture idiosyncratic data on maternal neurobiology and health in a more tailored manner. In the future, dense sampling models can integrate environmental contexts—such as fluctuations in relationship quality and trends in financial security—to further enrich models of women’s neurobiology and mental health during pregnancy.

## 7. Conclusions

Maternal brain literature has established that mothers experience profound neurocognitive and mental health changes during the perinatal period. While research on the maternal brain is still in its infancy, now is the time to consider the importance of examining individual differences in outcomes, which is facilitated through understanding the impact of environmental context on neurobiological adaptation and mental health during this period. Considering how environmental context shapes women’s neurobiology and mental health during the perinatal period has clear implications, especially on a policy level. Public policies that improve mothers’ and fathers’ access to paid family leave may improve their ability to seek mental health care during the perinatal period [62,63]. As discussed in this narrative review, examining pregnancy as a time of vulnerability to environmental contexts—especially those related to interpersonal relationships and physical resources—will help identify modifiable targets for improving mothers’ neurobiology and mental health.

## Data Availability

Not applicable.
